# Microperimetric Biofeedback Training After Successful Inverted Flap Technique for Large Macular Hole

**DOI:** 10.3390/jcm9020556

**Published:** 2020-02-18

**Authors:** Giancarlo Sborgia, Alfredo Niro, Tiziana Tritto, Valeria Albano, Luigi Sborgia, Alessandra Sborgia, Rossella Donghia, Ermete Giancipoli, Marco Coassin, Valentina Pastore, Gianluigi Giuliani, Umberto Lorenzi, Mario R. Romano, Francesco Boscia, Giovanni Alessio

**Affiliations:** 1 Department of Medical Science, Neuroscience and Sense Organs, Eye Clinic, University of Bari, 70124 Bari, Italy; gcsborgia@hotmail.it (G.S.); tiziana.tritto@gmail.com (T.T.); valeria.albano12@gmail.com (V.A.); luigi.sborgia@uniba.it (L.S.); valentinapastore@hotmail.it (V.P.); giuliani_gianluigi@hotmail.it (G.G.); francescoboscia@hotmail.com (F.B.); giovanni.alessio@uniba.it (G.A.); 2 Eye Clinic, Hospital “S. G. MOSCATI”, A.S.L. Taranto, 74010 Statte, Taranto, Italy; alessandrasborgia@yahoo.it; 3 National Institute of Gastroenterology “S. de Bellis” Research Hospital, 70013 Castellana Grotte, Bari, Italy; rossydonghia@gmail.com; 4 Eye Clinic, Hospital “V. FAZZI”, A.S.L. Lecce, 73100 Lecce, Italy; ermete.giancipoli@gmail.com; 5 Ophthalmology, University Campus Bio Medico of Rome, 00128 Roma, Italy; marcocoassin@hotmail.com; 6 Department of Ophthalmology, University Hospital of Rouen, 76000 Rouen, France; umbertolorenzi.oph@gmail.com; 7 Department of Ophthalmology, Humanitas University, 20090 Pieve Emanuele, Milan, Italy; mario.romano.md@gmail.com

**Keywords:** large macular hole, inverted flap technique, microperimeter, biofeedback, retinal sensitivity, fixation

## Abstract

**Background:** Despite the high closure rate of large macular hole (LMH) after surgery, visual recovery is often worse than expected. Microperimetric biofeedback can improve visual function in macular pathologies. We evaluated the efficacy of biofeedback on macular function after successful inverted flap technique for LMH. **Methods:** In this prospective comparative study, 26 patients after LMH surgical closure were enrolled. The whole sample was equally divided into two groups. In Group 1 (trained), patients underwent a double cycle of microperimetric biofeedback, using structured light stimulus plus acoustic tone; in Group 2 (control), patients underwent scheduled visits. We analyzed visual acuity, retinal sensitivity at central 12° (macular sensitivity, MS) and 4° (central macular sensitivity, CMS), and fixation stability over twelve months. **Results:** Visual acuity improved mainly in the trained group, without any significant differences between the groups (*p* > 0.05). Only after training did MS significantly improve (*p* = 0.01). CMS more significantly improved in the trained (*p* < 0.001) than the control group (*p* < 0.01) (Group 1 vs. 2, *p* = 0.004). Only in the trained group did fixation significantly improve (3 months, *p* ≤ 0.03; 12 months, *p* ≤ 0.01). An equality test on matched data confirmed a greater significant improvement of CMS (*p* ≤ 0.02) at all follow-up and fixation (*p* ≤ 0.02) at last follow-up after training. **Conclusions:** Microperimetric biofeedback consolidates and increases the improvement of retinal sensitivity and fixation gained after successful inverted flap technique.

## 1. Introduction

Idiopathic large macular hole (LMH) leads to a displacement of photoreceptors in the fovea, causing metamorphopsia and a severe visual acuity loss [1].

The inverted inner limiting membrane (ILM)-flap technique has become one of the most effective procedures, with more than 90% of closure rate of LMH [2,3,4,5].

Despite the high closure rate, not all previous reports made show significant functional improvement, and visual recovery is often worse than expected [6,7,8].

The inverted ILM-flap technique, promoting the closure of macular hole by the gliosis process controlled by Müller cells [9,10], could further hinder inner and outer retinal layers recovery with negative functional effects. Moreover, visual acuity recovery does not reveal many other functional changes related to macular hole and surgical maneuvers. Preoperative loss of macular integrity and morphologic modifications after surgery change retinal sensitivity and fixation behavior, measured by microperimetry (fundus-related perimetry), that play an important role in visual performance and could have a predictive role on visual acuity recovery [11,12,13,14,15]. If macular surgery could attempt to control morphologic changes over months and related functional recovery, patients usually tend to realize mechanisms of adaptation of visual system, as relocating the fixation at an extrafoveal site called preferred retinal locus (PRL) spontaneously, to bypass central scotoma [16,17,18,19]. Spontaneous retinal location of PRL is not always optimal for best visual performances [20], as it may be in far eccentric locations or in locations with low sensitivity [21]. Moreover, the eccentric fixation stability is worse than foveal fixation [22,23], and poor fixation stability is correlated with poor visual acuity [17,24,25,26]. Patients adapt typically within six months to using an eccentric locus for fixation, but the effective use of PRL requires extensive training [16], so neuro-psychology rehabilitative procedures, as microperimetric biofeedback, training patients to perform eccentric viewing, can increase retinal sensitivity and stabilize fixation improving visual performance [16]. The microperimetry uses the cerebral plasticity and neurosensory adaptation capabilities to improve the visual performance [27]. Previous studies with biofeedback technique using acoustic tone demonstrated the possibility to improve visual function, reading speed, fixation behavior and retinal sensitivity in several macular pathologies, as age-related macular degeneration (AMD), macular hole, rhegmatogenous retinal detachment, and macular dystrophy [27,28,29,30,31,32,33]. Moreover, a flickering visual stimulus is useful in the rehabilitation of patients with low vision at the late stage of AMD [34]. Acoustic stimulus combined to a standardized and structured light stimulus to perform biofeedback could seem more effective on retinal sensitivity and fixation stability [34,35]. In only two papers, the training was performed with more sessions per week, followed by a single session at each follow-up time point [30,36]. However, in one paper, the results were influenced by heterogeneity of the pathologies of study population [30], and in the other one by the limited significance of a case report [36]. In this study, we evaluated the efficacy of a standardized microperimeter biofeedback training, using a combination of visual and acoustic stimuli on the visual performance in patients with successful closure of LMH after inverted ILM-flap technique.

## 2. Materials and Methods

**Figure 1:**
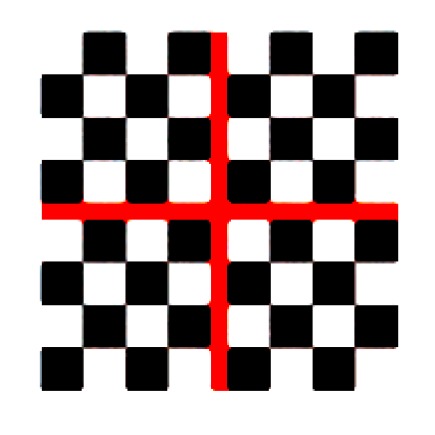
During each training session, the fixation target was replaced by a flickering structured pattern composed by a black-and-white reversal chessboard with a central red cross, which covered 8° of retina and whose elements had a size of about 0.5°.

A single-center prospective, comparative, non-randomized study was performed on 26 consecutive patients underwent successful 27-gauge vitrectomy with inverted ILM-flap technique for idiopathic LMH (diameter > 400 micron). In all cases, surgery was performed in the Eye Clinic of University of Bari, Bari, Italy, between April 2017 and April 2018. All the surgeries were performed by the same well-experienced retinal specialist (GS). Inclusion criteria were patients with idiopathic LMH in whom closure of LMH was confirmed with optical coherence tomography (OCT) at 1 month after surgery, best corrected visual acuity (BCVA) was better or equal to 1 LogMAR after surgery, and patients agreed with the MP-1 Microperimeter training and were followed up for 12 months. Exclusion criteria were the presence of ocular comorbidity or surgical complications that could affect visual performance. BCVA measured by an Early Treatment Diabetic Retinopathy Study (ETDRS) chart, was converted into LogMAR notation for statistical analysis. Macular sensitivity and fixation stability were evaluated by MP-1 microperimeter (MP-1, Nidek Technologies, Padova, Italy). The MP-1 provides a 45° non-mydriatic view of the fundus, with automated correction for eye movements. We performed microperimetry under room dim-light condition. MP-1 uses a background luminance of 10 cd/m^2^, maximum stimulus intensity of 125 cd/m^2^, stimulus size of 0.11–1.73 degrees (Goldmann I-V), white stimulus color, 0–20 dB dynamic range. Sensitivity was measured across a 45-point grid centered on the fovea, using pattern Macula 12°- 0dB. At each point in the grid, sensitivity was measured for a white stimulus 0.438 in diameter (Goldmann size III) presented for 200 ms against a mesopic background (1.27 cd/m^2^). Threshold at each point was determined by using a 4–2 staircase. The ‘‘follow-up’’ feature of the MP-1 was used to enable sensitivity measurements at the same retinal locations across all visits. Mean macular sensitivity (MS), the mean of all 45 loci in the central 12^°^ (1^°^ = 300μm), and mean central macular sensitivity (CMS), the mean sensitivity of the central 13 loci (enclosed by a circle with a 4° diameter), were recorded. Fixation stability was recorded, using the MP-1 during the light sensitivity examination. The standard target was represented by a red cross with an arm extension of 1°, but it was increased to ≥ 2° if patients were not able to see it. PRL is defined as an area that contains the center of a target image for over 20% of a fixation interval [16]. The bivariate contour ellipse area (BCEA) values were applied to obtain a quantitative measure of fixation stability in the area of eccentric PRL. BCEA is performed by plotting the position of each fixation on Cartesian axes and calculating the area of an ellipse that encompasses a given percentage of fixation points. The measure is based on the values of the standard deviations of the horizontal and vertical eye movements during fixation [37,38]. We analyzed BCEA, which contains 68.2%, 95.4%, and 99.6% of fixation points. Examination started after a 2 min demonstration pretest, to avoid a learning effect. Background luminance was 1.27 cd/m^2^. An auto-tracking system calculates the horizontal and vertical shifts relative to the reference during the examination recording the area of fixation. Examinations requiring longer than 15 min were excluded from the study. After recording baseline BCVA, MS, CMS, and BCEA at 1 month from surgery and after each patient signed the informed consent form, they were assigned into Group 1 or Group 2. In Group 1, all the patients underwent the same biofeedback rehabilitation protocol, using MP-1 Microperimeter. The first cycle consisted of 12 training sessions, with each one lasting 10 min, three times a week. After 6 months, the patients underwent second cycle, consisting of 4 training sessions, once a week. During each session, patients were asked to move the eye according to an audio feedback and a standardized, structured, and flickering light stimulus (a superimposed checkboard pattern with low spatial black/white elements with a size of about 0.5° onto fixation target, Figure 1) which advised patients when they were getting closer to the selected PRL. The frequency of the MP-1 auditory signal increased with the approach of fixation toward the PRL. Patients tried to focus on the fixation target according to the sound that became continuous when fixating with the PRL. Simultaneously the flickering structured pattern was projected on the PRL instead of the fixation target. As the patient was fixating with the PRL, the fixation target was replaced by the superimposed checkboard pattern with a central red cross. A 20 Hz of frequency for the flickering checkerboard was used. The fixation on the PRL had to be maintained as long as possible. (Video S1). The best PRL to be trained should have been located as close as possible to the scotoma, on the superior retinal field, with an appropriate retinal sensitivity to ensure the reinforcement of fixation, as demonstrated by Nilsson [39]. The best PRL was chosen by the ophthalmologist, preferring the PRL naturally developed by the patient within an area of fair retinal sensitivity and considered suitable for training. The fellow eye was occluded by using a simple eye-patch. All functional tests were performed with the patient’s best correctable prescription employed. All patients in Group 2 were followed up with standard care. They underwent a complete ophthalmologic examination, including visual acuity measurement, OCT, and microperimetry. We investigated all patients at 1 month post-surgery, as baseline time point, and at month 3,6, and 12. All data were collected by the same two operators (TT and VA).

The study was approved by our Institutional Review Board (IRB) (Registration number: 001/17; date: 09 January 2017, Eye Clinic, Department of Medical Science, Neuroscience and Sense Organs, University of Bari, Bari, Italy) and adhered to the tenets of the Declaration of Helsinki. An IRB approved informed consent was obtained from all patients.

### Statistical Analysis

Statistical analysis was based on all patients included in the study. Mean and standard deviation for continuous variables and relative frequency for categorical were used as indices of centrality and dispersion of the variable distribution.

For testing the difference between the two groups, the non-parametric test as Chi-square and Wilcoxon rank-sum test, were used.

The test of equality for matched data was used to compare the difference between pairs of observation in the control group and into the trained group.

When testing null hypothesis of no association, the probability level of α error at two-tailed was ≤0.05. All the statistical computations were made by using StataCorp, 2015, Stata Statistical Software: Release 14. College Station, TX: StataCorp LP.

## 3. Results

Twenty-six patients who underwent successful surgery with inverted ILM-flap technique for LMH were enrolled. Patients were divided into two groups of 13 subjects. In Group 1, the patients underwent biofeedback rehabilitation training, while in Group 2, patients were followed up with standard care. All patients completed the one-year follow-up visit.

Overall, there were 15 males and 11 females. Overall mean age was 63.8 ± 6.9 years. The age of the patients ranged from 51 to 76 years in Group 1 and 51 to 72 years Group 2. There was no statistical difference in demographic, pre-surgical anatomic (lens status, axial length, and macular hole minimum diameter), and functional parameters (BCVA, MS, CMS, and BCEA) between the groups (Table 1).

**Figure 2:**
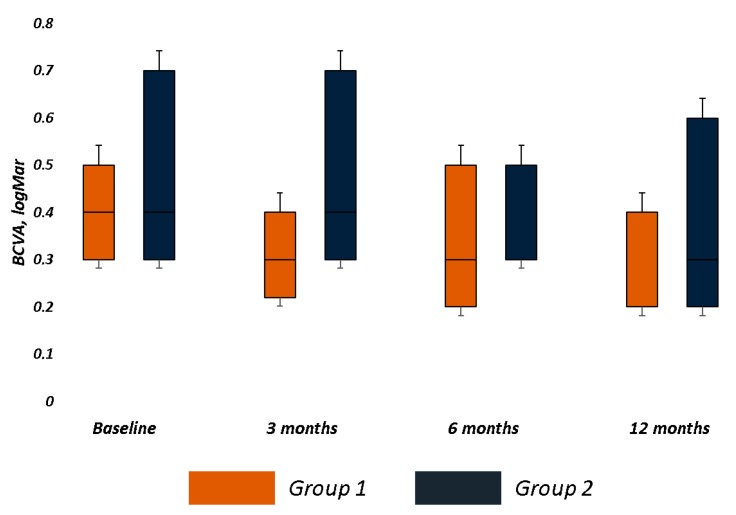
Best Corrected Visual Acuity (BCVA) changes over follow-up in both groups. The bar chart was built on the first quartile, median, and third quartiles of the dataset. Visual acuity (LogMAR) not significantly improved in trained (Group 1) and control (Group 2) groups. Major visual acuity improvement was achieved in trained group (0.12 LogMAR) vs. control group (0.06 LogMAR) at last follow-up.

Group 1 had better baseline functional parameters and a smaller fixation plot than Group 2, though there was no statistical difference between the groups for all functional parameters (Table 2). Although visual acuity had no significant improvement in each group and no significant difference between the groups at each follow-up, we recorded a mean improvement of 0.12 LogMAR and 0.06 LogMAR in Group 1 and 2, respectively (Table 2 and Figure 2).

**Figure 3:**
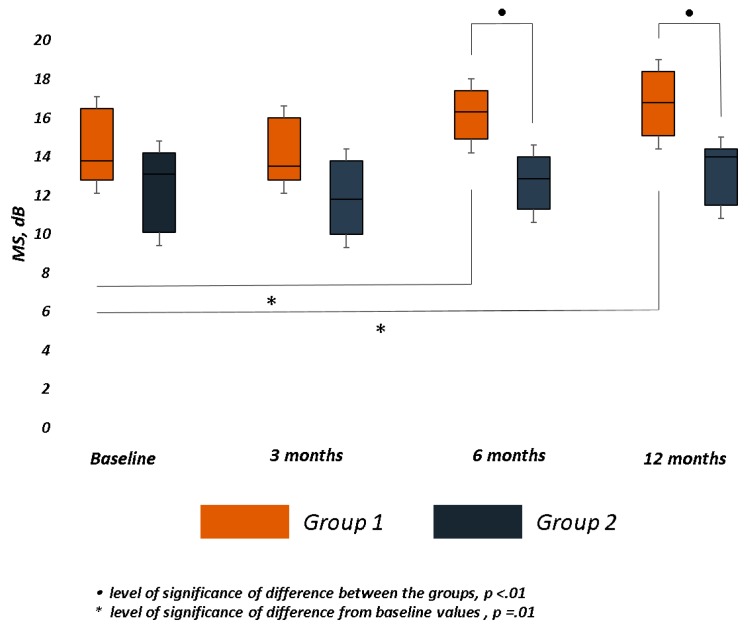
Macular Sensitivity (MS) changes over follow-up in both groups. The bar chart was built on the first quartile, median, and third quartiles of the dataset. Retinal sensitivity at central 12° (MS), measured in decibels (dB), significantly improved only in Group 1 at 6 and 12 months. At the same time points, the difference between the groups was statistically significant.

Mean MS significantly improved (*p* = 0.01) at month 6 and 12 in Group 1, while no significant improvement was recorded in Group 2 over follow-up. A significant difference between the groups was observed at month 6 (*p* = 0.004) and 12 (*p* = 0.008). (Table 2 and Figure 3).

**Figure 4:**
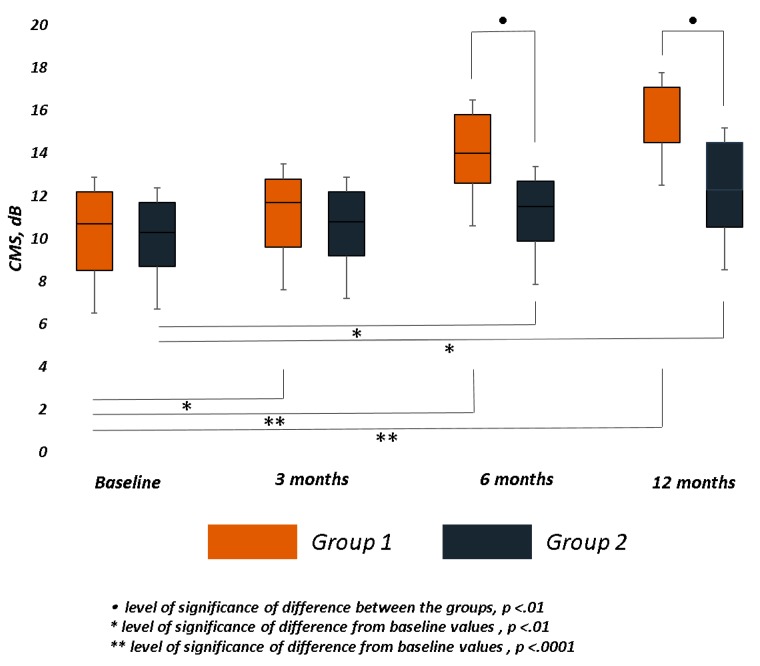
Central Macular Sensitivity (CMS) changes over follow-up in both groups. The bar chart was built on the first quartile, median, and third quartiles of the dataset. Retinal sensitivity at central 4° (CMS), measured in decibels (dB), significantly improved in both groups, mainly in trained group (Group 1). At 6 and 12 months, the difference between the groups was statistically significant.

In Group 1, the mean CMS significantly improved at all follow-ups (3 months, *p* < 0.01; 6 months, *p* < 0.001; 12 months, *p* < 0.001), while in Group 2, it significantly improved (p< 0.01) only at 6 and 12 months. A significant difference between the groups was observed at month 6 (*p* = 0.004) and 12 (*p* = 0.004) (Table 2 and Figure 4).

**Figure 5:**
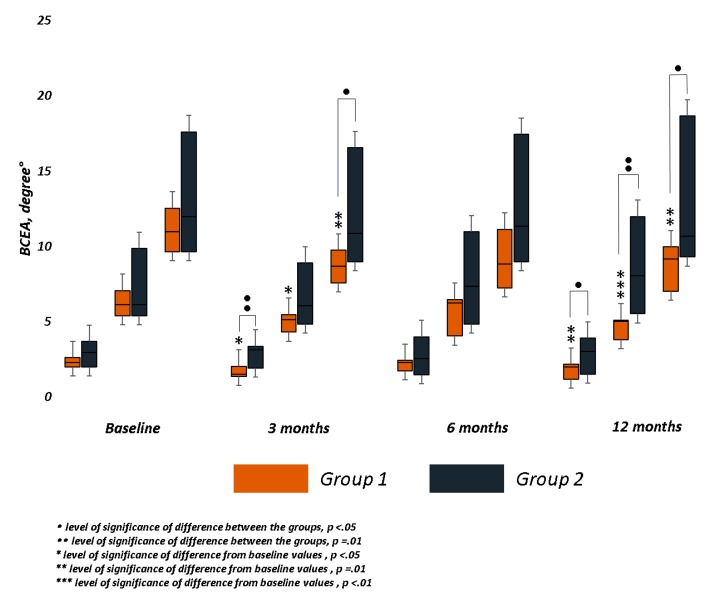
Fixation stability changes the Bivariate Contour Ellipse Area (BCEA, degree°) values were applied to obtain a quantitative measure of fixation stability. We analyzed the BCEA, which contains 68%, 95%, and 99% of fixation points. Only in the trained group (Group 1) did BCEA significantly decrease at all fixations points immediately after each training cycle, at 3 and 12 months. At the same follow-up, the differences between the groups were significant.

Only in Group 1 BCEA at three different ellipses area significantly decreased at month 3 (68.2%, *p* = 0.03; 95.4% *p* = 0.03; 99.6% *p* = 0.01) after first cycle of biofeedback and at month 12 (68.2%, *p* = 0.01; 95.4% *p* < 0.01; 99.6% *p* = 0.01) after second cycle of training. In Group 2, no significant reduction of fixation cloud at three different areas was recorded over follow-up. A significant difference between the groups was observed in BCEA 68.2% (*p* = 0.01) and 99.6% (*p* = 0.04) at month 3, and in BCEA 68.2% (*p* = 0.03) and 95.4% (*p* = 0.01) at last follow-up (Table 2 and Figure 5).

Comparing the difference between pairs of observation in the control group and in the biofeedback group for matched data, the difference between baseline BCVA and BCVA at each follow-up was not statistically significant (*p* > 0.05) in both groups. The difference between baseline BCEA and three- and six-month BCEA at all ellipses areas was not statistically significant (*p* > 0.05) in both groups. The difference between baseline BCEA and 12-month BCEA at all ellipses areas (68.2%, *p* = 0.02; 95.4% *p* = 0.003; 99.6% *p* = 0.02) was statistically significant only in the trained group. The difference between baseline MS and three- and six-months MS was not statistically significant (*p* > 0.05) in both groups, while comparing baseline MS and 12-month MS, the difference was significant in Group 1 (*p* = 0.04) and in Group 2 (*p* = 0.02). In Group 1, the difference between baseline CMS and CMS at all time points was significant (3 months, *p* = 0.02; 6 months, *p* = 0.003; 12 months, *p* < 0.001). In Group 2, the difference between baseline CMS and CMS was significant only at month 6 (*p* = 0.02) and 12 (*p* = 0.02).

## 4. Discussion

The influence of inverted ILM-flap technique on functional recovery was previously analyzed to understand whether visual recovery is affected by the flap over the hole. Indeed, the flap should provide an environment to instruct the photoreceptors in order to assume correct position during the reconstruction process and finally to improve visual acuity [2]. In line with previous papers [2,3,4,5,7,15,40,41], in both groups of this study, mean visual acuity improved after ILM-flap inversion. In particular, as we previously observed, a significant improvement of functional parameters as visual acuity, retinal sensitivity, and fixation was achieved as early as one month after surgery [15], so we considered that time point as baseline in this study. We first performed a biofeedback training on patients who underwent successful ILM-flap technique for LMH.

The biofeedback can be defined “as a technique able to increase the capacity of a person in controlling the voluntary physiological functions through the use of instruments that make its variations perceptible” [30]. Through the biofeedback the patient is trained to improve the use of visual nervous circuits not affected by the pathological process. In particular, the MP-1 biofeedback rehabilitation relocates the PRL to a healthy site on the retina, leading to improved function.

The acoustic biofeedback probably improves communication between intraretinal neurons, as well as retina–brain supporting a “remapping phenomenon” [27,28]. Ueda-Consolvo et al. applied an audio feedback training in eyes with poor functional recovery after traditional macular hole surgery. The PRL was chosen within central 2 degrees, and a 10 min training was repeated at least three times within three months. After training, patients demonstrated a significant increase in visual acuity, whereas fixation stability did not change significantly [31].

A flickering pattern stimulus has already been used for the treatment of hemianoptic visual field [42,43] and in patients affected by retinitis pigmentosa [44]. Pattern stimulation is composed by significant recognition shapes that could improve intraretinal integration processes, retina–brain transmission and stimulus recognition. Structured stimuli, together with the acoustic stimuli, could increase the function of PRL, as previously observed [34,35].

Thus, we used a combined approach, including visual and acoustic stimuli, and analyzed the effects on functional outcomes as visual acuity, retinal sensitivity, and a quantitative parameter of fixation behavior, as BCEA, comparing the results with a control group underwent common care strategy.

We did not find any significant differences in demographic and baseline data between the groups, and we considered this as a point of strength of the study. Moreover, presurgical parameters such as MH size, MS, and BCEA, which have a predictive role on visual acuity recovery [15], were not significantly different between the groups.

Visual acuity did not significantly improve over follow-up in both groups, although, in the biofeedback group, the 12-month mean visual acuity improvement was 0.12 LogMAR versus 0.06 LogMAR in the control group. The limitation in visual acuity improvement could be related to the small sample size and the incomplete recovery of outer retinal layers at the foveal site after macular hole closure [37], as we previously observed [15]. Moreover, out of central 2 degrees (1° = 300 μm), where PRL was frequently localized, we know a physiological fall down of visual acuity.

We also focused on the changes of MS and CMS, which represent the mean sensitivity within the central 12° and 4° of the macula, respectively. Previous papers showed that both MS and CMS improved after surgery [15]. However, MS and CMS more significantly improved in the biofeedback group than the control group over long follow-up. In both groups, MS recorded a mild reduction at first follow-up, followed by a more clinically significant improvement in the trained group at 6 and 12 months, when the difference between the groups was significant.

CMS incrementally improved from the first follow-up, with a total mean gain of 5.54 and 2.47 dB in Group 1 and 2, respectively. The difference of central sensitivity between the groups was significant at 6 and 12 months. The comparison between pairs of observation for matched data about sensitivity revealed significant differences respect to baseline CMS values at different follow-up, mainly in the trained group.

The similar trend of sensitivity observed in both groups could be related to the anatomical recovery after surgery, in particular due to the early and reversible functional damages on inner retinal layers due to ILM peeling at macular site [15,45,46,47] and the viability of detached photoreceptors at the edges of the hole within the foveal site [48,49]. Nonetheless, the biofeedback training significantly increased the improvement in retinal sensitivity, and probably the repetition of the training during the follow-up could consolidate the positive trend.

Fixation stability is another important functional parameter to be analyzed after macular hole surgery, probably more than fixation location that could already be naturally and irreversibly relocated out of the foveal site. Indeed, these patients usually tend to realize mechanisms of adaptation of visual system, as relocating the fixation to an extrafoveal PRL spontaneously [16,17,18,23,29].

In the biofeedback group, BCEA changed with a similar trend at 68.2%, 95.4%, and 99.6% of fixation points. We observed a significant reduction in the dimension of the cloud of the fixation points at month three after first cycle of training followed by a mild increase at six months and a significant reduction of all ellipses after the second cycle of training. Conversely, in the control group, we observed a mild impairment of fixation stability at last follow-up. Immediately after the first and second cycle of training, we observed significant differences between the groups at different area of fixation points. Moreover, significant differences between pairs of observation on BCEA for matched data, exactly baseline versus last follow-up values, were observed in the trained group. The improvement in the fixation stability gained with the training is related to the increase of the number of correct fixation saccades and to the time in which the fixation target falls on the PRL. We observed a “learning effect” in using eccentric fixation at PRL after each training cycle. Cerebral plasticity plays an important role in these results. Andrade et al. have shown that the cortical neurons related to the damaged retina do not remain inactive but become selective to stimuli originating in other area of the retina, often surrounding the lesion, as the PRL [50]. Biofeedback helps the brain to detect an efficient PRL. Thus, a fast redistribution of receptive fields, related to the area of the lesion, firstly occurs, followed by a long-term reorganization that leads to the final configuration of cortical receptive fields [50]. We speculated that the temporary effect on functional parameters after first cycle of biofeedback needed to be reinforced with other training sessions over follow-up. The repeated and persistent training practice of a searching movement toward PRL could result in lasting and consolidate physiological changes in motor neural networks. Another aspect to be considered is the combination of an acoustic tone and a structured light stimulus, which demonstrated better functional recovery on visual acuity, retinal sensitivity, and fixation stability compared to acoustic tone alone performing biofeedback training [34,35]. The audio feedback can help the brain to fix the PRL by increasing the attentional modulation, while the structural stimulus involves visual receptive fields that are highly sensitive to medium spatial frequencies [4,23]. However, previous papers on combined stimulation considered different macular diseases, not including macular hole, small population, and a not well-defined protocol of rehabilitation. Therefore, as points of strength of this study, we highlight the well-defined study population, the absence of significant differences between the groups about pre-surgical and baseline parameters, the standardized protocol of rehabilitation, the prospective nature of the study, and the long-term follow-up.

Limitations of the study include the small sample size, the absence of analysis of outer retinal layers features at OCT scans related to functional changes, the absence of analysis of vision-related quality of life changes after rehabilitation training, and the measurement error or intrinsic variability of microperimetric test. Factors acting on test variability are related to the patient’s compliance and to the examiner and/or instrument. In regard to the instrument, it should be mentioned that the eye-tracker system is not able to ensure the same accuracy of analysis between foveal and macular sites, the “point to point” overlapping error (0.5° to 2°) when “Follow-up” program is used, the “4-2 strategy” which can extend the duration of the test, the “ceiling effect” of MP-1, meaning the tendency to accumulate responses at the highest limit of the sensitivity threshold, and the size of the given stimulus (Goldmann III, 4 mm² area, 26 min diameter of arc, or 0.4°) that, because of “spatial summation”, involves more photoreceptors which converge centrally on a single ganglional cell. In conclusion, microperimetric biofeedback, probably improving intraretinal and retina–brain connections, can increase retinal sensitivity and stabilize fixation faster than common care strategy after successful inverted ILM-flap for LMH. Further research in studying the effect of this rehabilitation training on daily activities are required

## Figures and Tables

**Table 1:** Demographic and presurgical data for both groups.

Parameters ^*^	Group 1 (Biofeedback Group) n = 13	Group 2 (Control Group) n = 13	p^§^
Age, years			0.69
Mean (±SD)	62.7 ± 7.68	63,8 ± 6.16	
Range	51–76	51–72	
Gender			
M	8 (61.54)	7 (53.84)	0.69^^^
F	5 (38.46)	6 (46.15)	0.61
Axial length, mm			0.21
Mean (±SD)	23.73 ± 1.37	23.00 ± 0.88	
Range	21.56–26.5	21.12–24.04	
Lens Status			
Phakic	6 (46.15)	5 (38.46)	0.61
PseudoPhakic	7 (53.84)	8 (61.54)	0.69^^^
MH size, µm			0.78
Mean (±SD)	483.3 ± 52.5	489.7 ± 73.3	
Range	400–558	405–641	
BCVA, LogMAR			0.71
Mean (±SD)	0.97 ± 0.19	1.05 ± 0.22	
Range	1.4–0.5	1.4–0.9	
MS, dB			0.08
Mean (±SD)	13.55 ± 2.34	11.38 ± 3.15	
Range	9.1–15.8	3.1–15.8	
CMS, dB			0.24
Mean (±SD)	9.05 ± 2.24	8.01 ± 2.25	
Range	5.5–12.4	1.9–10.5	
BCEA, degree^2^			
Mean (±SD)			
at 68%	4.03 ± 2.64	5.03 ± 2.92	0.38
at 95%	10.40 ± 6.76	12.49 ± 6.60	0.43
at 99%	18.59 ± 12.16	21.04 ± 10.90	0.5

* All values: means ± standard deviations as continuous; frequencies and percentage (%) as categorical. Abbreviations: SD, standard deviation, MH, macular hole; IOP, intraocular pressure; BCVA, best corrected visual acuity; LogMAR, Logarithm of Minimum Angle of Resolution, MS, macular sensitivity; dB, decibel; CMS, central macular sensitivity; BCEA, biavariate contour ellipse area; § Wilcoxon rank-sum (Mann–Whitney) test; ^Chi-square.

**Table 2:** Baseline Best corrected visual acuity, retinal sensitivity and fixation behavior in both groups.

Parameters *	Group 1 (Biofeedback Group)	Group 2 (Control Group)	p⸹
BCVA, LogMAR (Baseline)			0.39
Mean (±SD)	0.40 ± 0.21	0.48 ± 0.24	
Range	0.9–0.09	1–0.20	
1° Cycle			
BCVA (3 months)	0.31 ± 0.15	0.45 ± 0.24	0.21
p^†^	0.06	0.26	
BCVA (6 months)	0.32 ± 0.18	0.43 ± 0.25	0.23
P	0.13	0.11	
2° Cycle			
BCVA (12 months)	0.28 ± 0.18	0.42 ± 0.26	0.17
P	0.06	0.08	
MS, dB (Baseline)			0.19
Mean (±SD)	14.31 ± 2.09	12.73 ± 2.72	
Range	12.2–17.1	9.1–16.8	
1° Cycle
MS (3 months)	13.95 ± 2.20	12.44 ± 2.53	0.12
P	0.64	0.68	
MS (6 months)	16.06 ± 1.81	13.11 ± 2.51	0.004
P	0.01	0.59	
2° Cycle			
MS (12 months)	16.48 ± 1.95	13.54 ± 2.63	0.008
P	0.01	0.14	
CMS, dB (Baseline)			0.64
Mean (±SD)	10.21 ± 2.28	9.64 ± 2.71	
Range	12.2–17.1	9.1–16.8	
1° Cycle			
CMS (3 months)	12.15 ± 2.82	10.59 ± 3.22	0.35
P	<0.01	0.2	
CMS (6 months)	14.82 ± 2.62	11.38 ± 3.29	0.004
P	<0.0001	<0.01	
2° Cycle			
CMS (12 months)	15.75 ± 2.15	12.11 ± 3.81	0.004
P	<0.0001	<0.01	
BCEA, degree° (Baseline) Mean ± SD			
68.2%	2.28 ± 0.69	3.01 ± 1.63	0.30
95.4%	6.04 ± 1.70	7.55 ± 4.19	0.44
99.6%	10.72 ± 3.03	12.36 ± 5.80	0.41
1° Cycle			
68.2% (3 months)	1.73 ± 0.50	2.74 ± 1.21	0.01
P	0.03	0.26	
95.4% (3 months)	4.76 ± 0.85	7.34 ± 4.24	0.10
p	0.03	0.42	
99.6% (3 months)	8.37 ± 1.44	12.25 ± 6.28	0.04
P	0.01	0.88	
68.2% (6 months)	2.09 ± 0.78	2.95 ± 2.10	0.33
P	0.55	0.77	
95.4% (6 months)	5.55 ± 1.96	8.43 ± 5.53	0.17
p	0.38	0.54	
99.6% (6 months)	9.29 ± 2.99	13.90 ± 9.93	0.19
p	0.16	0.62	
2° Cycle			
68.2% (12 months)	1.77 ± 0.56	3.31 ± 2.26	0.03
p	0.01	0.20	
95.4% (12 months)	4.49 ± 1.42	8.52 ± 5.04	0.01
p	<0.01	0.29	
99.6% (12 months)	8.06 ± 2.70	15.04 ± 10.55	0.07
p	0.01	0.19	

* All values: means ± standard deviations as continuous; p^†^, paired *t*-test for changes in each group; p⸹, Wilcoxon rank-sum (Mann–Whitney) test for changes among the groups; 1° Cycle, 12 Sessions/4 weeks of rehabilitation; 2° Cycle, 4 Sessions/4 weeks of rehabilitation; BCVA, Best Corrected Visual Acuity; LogMAR, Logarithm of Minimum Angle of Resolution; MS, Retinal Sensitivity; dB, decibel; CMS, Central Macular Sensitivity; BCEA 68.2%, 95.4%, and 99.6% Bivariate Contour Ellipse Area at 68%, 95%, and 99% of fixation points, respectively.

## Supplementary Materials

The following are available online at https://www.mdpi.com/2077-0383/9/2/556/s1. Video S1: Microperimetric biofeedback training session.
